# A cross-sectional study of career choice in obstetrics and gynecology and its influencing and discouraging factors among medical students in Eastern Saudi Arabia

**DOI:** 10.3389/fmed.2026.1795107

**Published:** 2026-02-19

**Authors:** Ayoob Lone, Humaira Zareen, Fahad Mohammed Alturkie, Ahmed Khalid Alnawah, Abdulaziz Shary Hadadi, Azam Tarek Alhedhod, Yousef Abdullah Aldreweesh, Amira Okud, Nawaf Al Khashram

**Affiliations:** 1 Department of Clinical Neuroscience, College of Medicine, King Faisal University, Al-Ahsa, Saudi Arabia; 2 Department of Obstetrics & Gynecology, College of Medicine, King Faisal University, Al-Ahsa, Saudi Arabia; 3 College of Medicine, King Faisal University, Al-Ahsa, Saudi Arabia; 4 Department of Biomedical Sciences, College of Medicine, King Faisal University, Al-Ahsa, Saudi Arabia

**Keywords:** career, gynecology, medical students, obstetrics, Saudi Arabia

## Abstract

**Background:**

According to the Saudi Commission for Health Specialties, the specialty of obstetrics and gynecology (OB/GYN) faces a growing gap between available residency positions and the number of applicants.

**Aim and objectives:**

The aim of the study was to investigate the medical students career choice in OB/GYN specialty and to identify the factors that influence or discourage their interest in pursuing this speciality as a future career.

**Materials and methods:**

This cross-sectional study invited 476 medical undergraduate students to complete an anonymous electronic survey that consisted of questions related to career intentions, opportunities, and attitudes of medical students toward OB/GYN and questions about factors that attract and discourage students from choosing OB/GYN as a career. We examined the statistical association between the categorical variables by using the Chi-square test. A binary logistic regression was performed to identify factors independently associated with increasing interest in OB/GYN.

**Results:**

The results of the present study indicated that 154 (32.3%) students were interested in OB/GYN. Sixty-one (19.2%) female participants reported OB/GYN as their first choice, while only 11 (6.9%) male participants expressed OB/GYN as their first choice (*p* < 0.01). The lack of interest is substantially higher among males (87.4%) than among females (57.8%). Interest increased with seniority, with interns showing the highest proportion selecting OB/GYN as their first choice (21.8%). Females perceived greater future opportunities in the specialty than males (40.7% vs. 27.7%, *p* = 0.02), and perceptions improved with academic seniority, with interns reporting the highest perceived opportunities (51.5%). In binary logistic regression, gender, university, and GPA were independently associated with interest. Male students had lower odds of interest compared with females (OR = 0.17, *p* < 0.01), and students from King Faisal University were less likely to be interested than those from Imam Abdulrahman Bin Faisal University (OR = 0.38, *p* = 0.01). GPA was a significant predictor: compared with students with a GPA of 4.51–5.00, those with a GPA < 1.5 (OR = 8.93, *p* = 0.01) and a GPA of 3.51–4.50 (OR = 1.94, *p* = 0.02) had higher odds of interest, while other GPA categories were not significant. Exposure-related factors, including OB/GYN rotations, faculty interaction, encouragement, and role models, were more frequently reported as attractive by females, whereas patient preference for female physicians, cultural expectations, stress, and anticipated impact on family life were prominent discouraging factors for males.

**Conclusion:**

Female medical students demonstrated greater interest in OB/GYN than males. Gender, academic performance, and institutional context were independently associated with career preference. Addressing modifiable barriers—particularly clinical exposure, mentorship, and cultural perceptions—may help reduce gender disparities and improve recruitment into OB/GYN.

## Introduction

Over the past three decades, several studies have reported fluctuating interest among medical graduates in pursuing obstetrics and gynecology (OB/GYN) as a career (1–7). Understanding the factors that influence medical students’ postgraduate specialty choices is essential for maintaining a balanced and sustainable national healthcare workforce (8). In particular, workforce planning requires alignment between the number of trained specialists and the growing clinical demand for services. This information is particularly significant in some parts of the world where students can select their specialization without taking a ranking exam, as is necessary in France and Belgium (8). Although OB/GYN is one of the main medical specialization that medical graduates might pursue as a future career, it is also one of the least appealing specialties to medical students (1, 9).

OB/GYN is often perceived as demanding due to heavy workloads, medico-legal pressures, and lifestyle challenges, contributing to declining interest in many countries (1, 5, 10). At the same time, population growth, rising maternal health needs, and increasing service expectations have led to a sustained and, in some regions, growing demand for obstetric and gynecological care. In the Gulf region, specialty choice is further shaped by cultural expectations, gender norms, clinical exposure, and anticipated work–life balance (9, 11–14).

In Saudi Arabia, this imbalance between workforce demand and trainee recruitment is becoming increasingly evident, with a growing mismatch between OB/GYN residency applicants and available positions, raising concerns about a potential recruitment crisis (3, 9). According to previous findings, gender consideration, lifestyle, remuneration, stress level, litigation, and interest in the field are key determinants of applicants for OB/GYN residency, yet several countries show differing ratios of the importance of various elements (1). Therefore, there is a concerning disparity between the number of applicants and the rising demand for obstetricians and gynecologists. This raises major questions about the kind of care that overworked and weary doctors in the field can offer, which could result in a higher incidence of problems, patient complaints and discontent, and potential medico-legal actions (3).

Several studies have been conducted nationally and internationally that explored the influencing factors in choosing OB/GYN as a career choice among medical students (4, 15–17). While a study in Taiwan identified clinical interest and sense of accomplishment as main motivators (1), these findings reflect Taiwan’s context and may not be generalizable to Saudi Arabia. Another study reported that female gender was the only significant factor associated with choosing OB/GYN specialty as a career, and male participants reported barriers preventing them from choosing this specialty (18). Similarly, a Swiss study found that experiential factors such as hands-on procedures, clinical enthusiasm, skill affinity, and gender strongly influenced OB/GYN career choice (8).

Within Saudi Arabia, only a small proportion of students rank OB/GYN among their top career choices—9.7% in Abu-Rafea et al. (3), and 8.8% as a first choice in Jazan—with female students more likely to pursue the specialty (9). Factors such as rotations, faculty interaction, procedural experience, cultural acceptance, and family expectations have been identified as enablers, while stress, workload, and medico-legal concerns act as barriers (3, 9, 19, 20).

## Rationale for the study

Obstetrics and gynecology plays a critical role in healthcare delivery, particularly in addressing maternal and reproductive health needs, yet interest in this specialty among medical students remains low worldwide, including in Saudi Arabia. Multiple factors influence specialty choice, such as perceived workload, work–life balance, stress, medico-legal risk, financial considerations, and intrinsic interest. Gender and sociocultural expectations are especially relevant in OB/GYN, as cultural norms in Saudi Arabia may facilitate female participation while creating perceived or practical barriers for male students. Although prior studies have explored determinants of specialty choice, findings are heterogeneous, and international evidence may not fully capture the Saudi context (3, 9). Given the growing gap between workforce demand and recruitment into OB/GYN, context-specific evidence is needed to understand how cultural, educational, and institutional factors shape medical students’ perceptions and career intentions. Therefore, this study seeks to identify the factors that influence or discourage medical students’ interest in pursuing OB/GYN as a future career within the Eastern Governorate of Saudi Arabia, with the goal of informing workforce planning and targeted educational strategies.

## Methods

### Study design

A descriptive, cross-sectional study was performed among medical students studying in public universities of Saudi Arabia. Data was collected between September 2025 and November 2025. This study was conducted in accordance with the Helsinki Declaration for human research, and Ethical clearance was obtained from the Deanship of Scientific Research, King Faisal University (KFU-REC-2023-ETHICS869). All study participants were informed about the goal and aim of the research, and the survey was conducted after getting signed consent from the participants.

### Setting

The study was conducted at two governmental universities of Saudi Arabia: King Faisal University, located in Al-Ahsa, and Imam Abdulrahman Bin Faisal University, located in Dammam. Both institutions are public universities governed and funded by the Saudi Ministry of Education, offering comprehensive undergraduate medical programs that include pre-clinical and clinical phases. At both universities, medical students progress through structured clinical rotations in various specialties, including OB/GYN, internal medicine, surgery, pediatrics, and family medicine. The OB/GYN rotation exposes students to inpatient and outpatient care, antenatal and postnatal management, labor and delivery, surgical procedures, and patient counseling. This clinical training is designed to provide students with foundational knowledge, practical skills, and professional competencies necessary to make informed decisions about their future specialty choice. These two universities were selected for this study because they represent typical academic and clinical environments in the Eastern Region, offering comparable curricula, structured exposure to OB/GYN, and access to diverse patient populations. The inclusion of both institutions enhances the generalizability of findings regarding medical students’ perceptions, motivations, and barriers associated with pursuing OB/GYN as a future career in Saudi Arabia.

### Study population

The medical students enrolled in Universities located in the Eastern Governorate of Saudi Arabia were included in our research. Approximately 3,100 medical students were enrolled in these universities. Out of 3,100 students, approximately 1800 students were from King Faisal University, and 1,300 medical students were enrolled from Imam Abdulrahman Bin Faisal University. Both universities follow a six-year Bachelor of Medicine and Surgery (MBBS) program, consisting of three preclinical years and three clinical years, and implement similar innovative teaching strategies designed to prepare competent future physicians. These strategies include integrated curricula, problem-based learning, early clinical exposure, and structured clinical rotations. The inclusion criteria were MBBS students, age >18 years and Saudi citizens. The study excluded those <18 years, non-Saudi, private college students, non-medical students, those unwilling to fill the questionnaire, and those who did not consent to participate.

### Sampling and sample size calculation

Participants were recruited using a probability -based stratified random sampling approach. To ensure that the sample accurately reflected the sociodemographic characteristics of the target population, students were first stratified by academic year. Within each stratum, students were randomly selected using a randomization procedure, ensuring proportional representation of each academic level in the final sample. The sample size for this study was calculated using Slovin’s formula (21) with a confidence interval of 95% and a margin of error of 0.05. Based on this calculation, a total of 400 students were initially selected, and the sample was subsequently increased to 500 participants to enhance representativeness. Out of the 500 students approached, 476 completed the survey, yielding a response rate of 95.2%. Missing data from 24 participants were excluded from the analysis.

### Study tools

An adapted English version of two previously designed questionnaires was used to fulfil the study aim (9, 22). There were three sections in the questionnaire consists of 30 items; Five items for career intention, opportunities and attitude toward OB/GYN. Eighteen items for factors related to attraction and discouragement of the OB/GYN, and seven questions for demographic information.

### Career intention, opportunities and attitude towards OB/GYN

The first section of the questionnaire consisted of five items assessing participants’ career intentions, perceived training opportunities, and attitudes toward OB/GYN. Career intention to choose OB/GYN as a future specialty was assessed using one item, with responses rated on a 3-point Likert scale: 1 (first choice), 2 (second choice), and 3 (not interested). Participants who indicated that they were not interested in pursuing OB/GYN were subsequently asked an additional item regarding their alternative career interests. Perceived opportunities for training in OB/GYN were evaluated using one item rated on a 3-point Likert scale: 1 (good), 2 (limited), and 3 (not sure). Attitudes toward the potential separation of obstetrics and gynecology into distinct specialties were assessed using one item rated on a 3-point Likert scale: 1 (agree), 2 (do not agree), and 3 (not sure). Finally, the perceived impact of splitting obstetrics and gynecology on the attractiveness of the specialty was assessed using a single multiple-choice item capturing students’ preference: (1) preference to concentrate on obstetrics, (2) preference to concentrate on gynecology, (3) preference to practice both obstetrics and gynecology (i.e., not favoring a split), and (4) no interest in the specialty regardless of division.

### Attracting and discouraging factors

To assess factors influencing attraction to or discouragement from obstetrics and gynecology (OB/GYN), an 18-item scale adapted from previous studies was employed (22). The items covered educational exposure and mentorship (rotation through OB/GYN during medical school, presence of role models, faculty interaction, faculty encouragement, and prior interaction with OB/GYN residents); specialty characteristics (opportunities to care for a generally healthy population, surgical opportunities, hands-on procedures, and the intellectual content of OB/GYN); socio-cultural influences (patients’ preference for a female physician and cultural expectations); workload and lifestyle considerations (length of residency, time demands, level of stress, and night duties); and personal and financial considerations (income prospects/financial opportunities, spouse or family opinion, and the perceived impact of an OB/GYN career on family life). Participants rated each item on a 3-point Likert scale ranging from 1 (discouraged), 2 (neutral), to 3 (attracted). Domain-specific and overall scores were calculated by summing the corresponding item responses, with higher scores indicating a more favorable perception of OB/GYN as a career choice. The internal consistency reliability (Cronbach’s alpha) of the measure used in the present study was 0.89.

### Demographical data

The demographic information, including age, gender, academic year, marital status, monthly income, GPA, and medical school attended, was collected using the questionnaire.

### Data collection

Before data collection, a pilot study was conducted with 30 students to confirm the reliability and validity of the questionnaire. Data from these students were not included in the final analysis. Trained medical students were recruited from both of the universities to reach the targeted sample. Following random selection, the selected students were contacted using institutional communication channels. Each academic batch maintains an official social network (WhatsApp) group used for academic communication. Group administrators were contacted only to facilitate dissemination of study information to the randomly selected students, and participants’ institutional email addresses were subsequently obtained. Before data collection, each student was contacted via email to determine his or her willingness to participate in the study. After obtaining written consent, the students were asked to complete an electronic survey (Google Form). Participation was entirely voluntary, and no incentives were offered.

### Statistical analysis

After verifying the quality and consistency of the data, they were coded and exported to the Statistical Package for the Social Sciences (IBM SPSS, version 27) for analysis. Prior to inferential analysis, the main outcome variables were assessed for normality using visual inspection (histograms and Q–Q plots) and Kolmogorov–Smirnov tests. Categorical variables were summarized as frequencies and percentages, and associations between them were assessed using the Chi-square test. Career preference for OB/GYN was dichotomized into “Interested” (first or second choice) and “Not Interested.” To identify factors independently associated with interest in OB/GYN, binary logistic regression was performed. Independent variables entered into the model included age group, gender, marital status, university attended, academic year, grade point average (GPA), and family income. Categorical predictors were entered using indicator (dummy) coding, with appropriate reference categories defined for each variable. Results were expressed as adjusted odds ratios (ORs) with 95% confidence intervals (CIs). A *p*-value of less than 0.05 was considered statistically significant.

## Results

### Participants characteristic

The present study invited 500 students studying in different medical colleges of the Eastern Province of Saudi Arabia. A total of 476 students (Male = 159; Females = 317) finally completed the questionnaire. The other 24 participants who were reluctant to respond to all items of the questionnaire were excluded. Table 1 shows that the majority of 71.4% participants were between 21 and 24 years old. Most of the participants, 371 (77.9%), were enrolled in King Faisal University, and 105 (22.1%) participants were enrolled in Imam Abdulrahman Bin Faisal University. The percentages of single and married participants were 81.3 and 18.7, respectively and GPA distribution indicated that 47.5% of students scored 4.51–5.00. Overall, the sample represented multiple academic years, genders, and institutional backgrounds, providing a diverse perspective on career preferences in OB/GYN.

**Table 1:** Demographic characteristics of study participants.

Characteristics	*N*	%
Gender		
Male	159	33.4
Female	317	66.6
Age		
<20 years	63	13.2
21–24 years	340	71.4
>25 years	73	15.3
Academic year		
Preparatory year	15	3.1
1st year	37	7.8
2nd year	69	14.5
3rd year	84	17.6
4th year	76	16.0
5th year	94	19.7
Intern	195	40.9
Medical school attended		
King Faisal University	371	77.9
Imam Abdulrahman Bin Faisal University	105	22.1
Marital status		
Single	387	81.3
Married	89	18.7
Monthly income (SAR)		
< 2000	243	51.1
2001–5,000	90	18.9
5,001–10,000	83	17.4
10,001–15,000	24	5.0
>15,001	36	7.6
GPA		
<1.5	12	2.5
1.51–2.50	20	4.2
2.51–3.50	94	19.7
3.51–4.50	124	26.1
4.51–5.00	226	47.5

### Career interest in OB/GYN

Table 2 presents the respondents’ interests in OB/GYN as a future career among medical students. Out of 476 students, 154 (32.3%) expressed interest in OB/GYN, with female students showing substantially higher interest than males (57.7% vs. 12.6%). Female students were also more likely to rank OB/GYN as their first choice (19.2% vs. 6.9%). Interest tended to be higher among older students (>25 years), intern students (final-year medical students completing clinical rotations in hospitals as part of their internship), and students from Imam Abdulrahman Bin Faisal University, while students in earlier academic years and from King Faisal University showed lower interest. Marital status, monthly income, and GPA showed minor variations, with married students and those with moderate GPAs slightly more inclined toward OB/GYN.

**Table 2:** Career intention, opportunities and attitude of medical students toward obstetrics and gynecology based on different demographic variables.

		Career intention			*p* value
		First choice	Second choice	Not interested	
		*N* (%)	*N* (%)	*N* (%)	
Gender	Male	11 (6.9)	9 (5.7)	139 (87.4)	0.00
	Female	61 (19.2)	73 (23.0)	183 (57.8)	
Age	<20 years	8 (12.7)	11 (17.5)	44 (69.8)	0.00
	20-25 years	43 (12.6)	55 (16.2)	242 (71.2)	
	>25 years	21(28.8)	16 (21.9)	36 (49.3)	
Academic year	Preparatory year	2 (13.3)	4 (26.7)	9 (60.0)	0.03
	Ist year	6 (16.2)	3 (8.1)	28 (75.7)	
	2nd year	13 (18.9)	9 (13.0)	47 (68.1)	
	3rd year	14 (16.6)	15 (17.9)	55 (65.5)	
	4th year	7 (9.2)	19 (25.0)	50 (65.8)	
	5th year	8 (8.5)	10 (10.6)	76 (80.9)	
	Intern	22 (21.8)	22 (21.8)	57 (56.4)	
Medical school attended	King Faisal University	39 (10.5)	61 (16.4)	271 (73.1)	0.00
	Imam University	33 (31.4)	21 (20.0)	51 (48.6)	
Marital status	Single	49 (12.7)	62 (16.0)	276 (71.3)	0.00
	Married	23 (25.8)	20 (22.5)	46 (51.7)	
Monthly income (SAR)	<2000	30 (12.3)	41 (16.9)	172 (70.8)	0.00
	2001–5,000	9 (10.0)	13 (14.4)	68 (75.6)	
	5,001–10,000	25 (30.1)	18 (21.7)	40 (48.2)	
	10,001–15,000	4 (16.7)	4 (16.7)	16 (66.6)	
	>150,001	4 (11.1)	6 (16.7)	26 (72.2)	
Grade point average	<1.5	9 (75.0)	0 (0.0)	3 (25.0)	
	1.51–2.50	3 (15.0)	5 (25.0)	12 (60.0)	0.00
	2.51–3.50	16 (17.0)	18 (19.2)	60 (63.8)	
	3.51–4.50	23 (18.5)	27 (21.8)	74 (59.7)	
	4.51–5.00	21 (9.3)	32 (14.2)	173 (76.5)	

		Opportunities			*p* value
		More	Less	Not sure	
		*N* (%)	*N* (%)	*N* (%)	
Gender	Male	44 (27.7)	36 (22.6)	79 (49.7)	0.02
	Female	129 (40.7)	61 (19.2)	127 (40.1)	
Age	<20 years	21 (33.3)	10 (15.9)	32 (50.8)	0.00
	20-25 years	111 (32.6)	75 (22.1)	154 (45.3)	
	>25 years	41(56.1)	12 (16.4)	20 (27.3)	
Academic Year	Preparatory year	5 (33.3)	2 (13.3)	8 (53.4)	0.04
	Ist year	14 (37.8)	4(10.8)	19 (51.4)	
	2nd year	20 (29.0)	13 (18.8)	36 (52.2)	
	3rd year	25 (29.8)	17 (20.2)	42 (50.0)	
	4th year	27 (35.5)	17 (22.4)	32 (42.1)	
	5th year	30 (31.9)	21 (22.3)	43 (45.8)	
	Intern	52 (51.5)	23 (22.8)	26 (25.7)	
Medical school attended	King Faisal University	120 (32.3)	76 (20.5)	175 (47.2)	0.00
	Imam University	53 (50.5)	21 (20.0)	31 (29.5)	
Marital Status	Single	128 (33.1)	80 (20.7)	179 (46.2)	0.00
	Married	45 (50.6)	17 (19.1)	27 (30.3)	
Monthly Income (SAR)	<2000	73 (30.0)	49 (20.2)	121 (49.8)	0.00
	2001–5,000	23 (25.6)	20 (22.2)	47 (52.2)	
	5,001–10,000	49 (59.0)	12 (14.5)	22 (26.5)	
	10,001–15,000	14 (58.3)	7 (29.2)	3 (12.5)	
	>150,001	14 (38.9)	9 (25.0)	13 (36.1)	
Grade point average	<1.5	10 (83.4)	1 (8.3)	1 (8.3)	0.01
	1.51–2.50	9 (45.0)	3 (15.0)	8 (40.0)	
	2.51–3.50	36 (38.3)	14 (14.9)	44 (46.8)	
	3.51–4.50	48 (38.7)	25 (20.2)	51 (41.1)	
	4.51–5.00	70 (31.0)	53 (23.4)	103 (45.6)	

		Attitude of splitting of obstetrics and gynecology			*p* value
		Agree	Don not agree	Not sure	
		*N* (%)	*N* (%)	*N* (%)	
Gender	Male	55 (34.6)	40 (25.2)	64 (40.2)	0.33
	Female	127 (40.1)	63 (19.9)	127 (40.0)	
Age	<20 years	21 (33.3)	11 (17.5)	31 (49.2)	0.00
	20-25 years	120 (35.3)	79 (23.2)	141 (41.5)	
	>25 years	41 (56.2)	13 (17.8)	19 (26.0)	
Academic Year	Preparatory year	4 (26.7)	2 (13.3)	9 (60.0)	0.23
	Ist year	14 (37.8)	5 (13.5)	18 (48.7)	
	2nd year	25 (36.2)	16 (23.2)	28 (40.6)	
	3rd year	30 (35.7)	18 (21.4)	36 (42.9)	
	4th year	28 (36.8)	18 (23.7)	30 (39.5)	
	5th year	29 (30.8)	25 (26.6)	40 (42.6)	
	Intern	52 (51.5)	19 (18.8)	30 (29.7)	
Medical school attended	King Faisal University	122 (32.9)	86 (23.2)	163 (43.9)	0.00
	Imam University	60 (57.1)	17 (16.2)	28 (26.7)	
Marital Status	Single	130 (33.6)	91 (23.5)	166 (42.9)	0.00
	Married	52 (58.4)	12 (13.5)	25 (28.1)	
Monthly Income (SAR)	<2000	83 (34.2)	59 (24.3)	101 (41.5)	0.00
	2001–5,000	31 (34.4)	13 (14.5)	46 (51.1)	
	5,001–10,000	46 (55.4)	18 (21.7)	19 (22.9)	
	10,001–15,000	11 (45.8)	3 (12.5)	10 (41.7)	
	>150,001	11 (30.5)	10 (27.8)	15 (41.7)	
Grade point average	<1.5	10 (83.4)	1 (8.3)	1 (8.3)	0.00
	1.51–2.50	8 (40.0)	3 (15.0)	9 (45.0)	
	2.51–3.50	33 (35.1)	17 (18.1)	44 (46.8)	
	3.51–4.50	56 (45.2)	17 (13.7)	51 (41.1)	
	4.51–5.00	75 (33.2)	65 (28.8)	86 (38.0)	

*p* < 0.05.

The percentage of perception of opportunities to train in OB/GYN is shown in Table 2. Among the 476 participants, 173 (36.3%) perceived adequate training opportunities in OB/GYN (Table 2). Perceived opportunities were more commonly reported by female students than males and were higher among older students (>25 years) and interns. In contrast, younger students (<20 years) and preparatory-year students were more likely to express uncertainty. Institutional variation was observed, with students from Imam Abdulrahman Bin Faisal University more frequently reporting perceived opportunities, whereas students from King Faisal University more often reported uncertainty. Married students and those with a moderate monthly income (5001–10,000 SAR) were more likely to perceive opportunities in OB/GYN, while unmarried students and those with lower income levels tended to be unsure. Similarly, students with an average GPA more frequently perceived opportunities, whereas those with GPAs between 2.51 and 3.50 were more likely to report uncertainty.

As shown in Table 2, agreement with splitting obstetrics and gynecology was more common among older participants (>25 years), whereas younger students (<20 years) more frequently reported uncertainty. Students from Imam Abdulrahman Bin Faisal University were more likely to support splitting OB/GYN, while those from King Faisal University tended to be uncertain. Married participants more often agreed with splitting the specialty, whereas unmarried students predominantly expressed uncertainty. Higher agreement was also observed among students with moderate monthly income (5001–10,000 SAR) and those with average GPA, while participants with lower income levels and GPAs between 2.51 and 3.50 were more likely to be unsure.

### Factors associated with interest in OB/GYN

Binary logistic regression was conducted to examine factors associated with medical students’ interest in OB/GYN as a career choice (first or second choice vs. not interested). Among the predictors included, gender, university attended, and GPA were significantly associated with career preference, while age group, marital status, academic year, and family income were not found to be significant. Results presented in Table 3 show that gender was a strong and statistically significant predictor of career preference. Male students had significantly lower odds of being interested in OB/GYN compared with female students (*B* = −1.76, OR = 0.17, 95% CI: 0.09–0.30, *p* < 0.01). Binary logistic regression was conducted to examine factors associated with medical students’ interest in OB/GYN as a career choice (first or second choice vs. not interested). Among the predictors included, gender, university attended, and GPA were significantly associated with career preference, while age group, marital status, academic year, and family income were not found to be significant. Results presented in Table 3 show that gender was a strong and statistically significant predictor of career preference. Male students had significantly lower odds of being interested in OB/GYN compared with female students (*B* = −1.76, OR = 0.17, 95% CI: 0.09–0.30, *p* < 0.01). This indicates that male students were approximately 83% less likely to express interest in OB/GYN than female students. The university attended was also significantly associated with career interest. Students from King Faisal University had lower odds of being interested in OB/GYN compared with students from Imam Abdulrahman Bin Faisal University (*B* = −0.97, OR = 0.38, 95% CI: 0.19–0.73, *p* = 0.01). Academic performance was significantly associated with interest in OB/GYN. Compared with students in the highest GPA category (4.51–5.00, reference), students with a GPA < 1.5 were much more likely to be interested in OB/GYN (*B* = 2.19, OR = 8.93, 95% CI: 2.07–38.45, *p* = 0.01). Additionally, students with a GPA of 3.51–4.50 also had significantly higher odds of interest (*B* = 0.66, OR = 1.94, 95% CI: 1.13–3.34, *p* = 0.01). The other GPA categories (1.51–2.50 and 2.51–3.50) were not significantly associated with OB/GYN interest.

**Table 3:** Binary logistic regression identifying predictors of OB/GYN career preference.

Variables	*B*	S.E.	Sig.	Exp(B)	95% Confidence Interval	
					Lower bound	Upper bound
Gender (ref: female)						
Male	−1.76	0.29	0.01**	0.17	0.09	0.30
Age (ref: >25 years)						
<20 years	−0.05	0.76	0.94	0.94	0.21	4.25
21–24 years	−0.56	0.51	0.26	0.56	0.20	1.54
Marital status (ref: Married)						
Single	−0.33	0.31	0.29	0.71	0.38	1.33
Monthly income (ref: >15,001)						
< 2000	0.28	0.42	0.50	1.32	0.57	3.03
2001–5,000	−0.41	0.50	0.41	0.66	0.24	1.77
5,001–10,000	0.32	0.53	0.54	1.38	0.49	3.90
10,001–15,000	0.14	0.61	0.81	1.15	0.34	3.84
University attended (ref: Imam Abdulrahman Bin Faisal University)						
King Faisal University	−0.97	0.33	0.01**	0.38	0.19	0.73
Academic year (ref: Intern)						
Preparatory year	−0.31	0.89	0.72	0.73	0.12	4.24
1st year	−0.23	0.75	0.75	0.79	0.18	3.47
2nd year	0.00	0.52	0.98	1.00	0.36	2.81
3rd year	0.53	0.46	0.25	1.70	0.68	4.26
4th year	0.32	0.46	0.48	1.38	0.55	3.43
5th year	−0.33	0.45	0.45	0.71	0.29	1.73
GPA (ref: 4.51–5.00)						
<1.5	2.19	0.74	0.01**	8.93	2.07	38.45
1.51–2.50	0.57	0.53	0.28	1.77	0.62	5.04
2.51–3.50	0.35	0.32	0.28	1.42	0.75	2.69
3.51–4.50	0.66	0.27	0.02*	1.94	1.13	3.34

**p < 0.01; *p < 0.05.

### Attracting and discouraging factors

As summarized in Table 4, attracting and discouraging factors for pursuing OB/GYN differed notably by sex. Among female students, key attracting factors included exposure during OB/GYN rotations, positive faculty interaction and encouragement, the presence of role models, and the intellectual content of the specialty. Sociocultural factors—particularly patient preference for female physicians and cultural expectations—also played an important role. In contrast, high perceived stress and demanding work schedules, including night duties, were the primary discouraging factors for female students.

**Table 4:** Attracting and discouraging factors of obstetrics and gynecology among medical students.

Factors	Gender	Attracted	Discouraged	Neutral	*p* value
		*N* (%)	*N* (%)	*N* (%)	
Rotation through OBGYN during medical school	Male	4	4	4	4
		57 (35.8)	39 (24.5)	63 (39.6)	0.00
	Female	159 (50.1)	50 (15.8)	108 (34.1)	
Presence of a role model in the OB/GYN specialty	Male	49 (30.8)	40 (25.2)	70 (44.0)	0.04
	Female	129 (40.7)	62 (19.6)	126 (39.7)	
Faculty interaction	Male	61 (38.4)	27 (17.0)	71 (44.6)	0.00
	Female	194 (61.2)	28 (8.8)	95 (30.0)	
Faculty encouragement	Male	67 (42.1)	20 (12.6)	72 (45.3)	0.05
	Female	168 (53.0)	25 (7.9)	124 (39.1)	
Previous interaction with OBGYN residents	Male	73 (45.9)	19 (11.9)	67 (42.1)	0.28
	Female	170 (53.6)	31 (9.8)	116 (36.6)	
Taking care of a healthy population	Male	94 (59.1)	15 (9.4)	50 (31.5)	0.03
	Female	182 (57.4)	18 (5.7)	117 (36.9)	
Patients’ desire for a female physician	Male	30 (18.9)	84 (52.8)	45 (28.3)	0.00
	Female	203 (64.1)	20 (6.3)	94 (29.6)	
Cultural expectations	Male	23 (14.5)	87 (54.7)	49 (30.8)	0.00
	Female	159 (50.2)	44 (13.9)	114 (35.9)	
Surgical opportunities	Male	72 (45.3)	23 (14.5)	64 (40.2)	0.04
	Female	140 (44.1)	57 (18.0)	120 (37.9)	
Hands-on procedures	Male	68 (42.8)	22 (13.8)	69 (43.4)	0.18
	Female	155 (48.9)	52 (16.4)	110 (34.7)	
Intellectual content of OB/GYN	Male	56 (35.2)	30 (18.9)	73 (45.9)	0.00
	Female	155 (48.9)	33 (10.4)	129 (40.7)	
Length of residency	Male	44 (27.7)	27 (17.0)	88 (55.3)	0.45
	Female	100 (31.5)	61 (19.2)	156 (49.2)	
Time demands	Male	38 (23.9)	41 (25.8)	80 (50.3)	0.22
	Female	92 (29.0)	92 (29.0)	133 (42.0)	
Level of stress	Male	42 (26.4)	46 (28.9)	71 (44.7)	0.02
	Female	85 (26.8)	127 (40.1)	105 (33.1)	
Night duties	Male	40 (25.5)	50 (31.4)	69 (43.4)	0.04
	Female	83 (26.2)	124 (39.1)	110 (34.7)	
Income prospects/ financial opportunities	Male	50 (31.4)	24 (15.2)	85 (53.4)	0.23
	Female	125 (39.4)	40 (12.6)	152 (48.0)	
Spouse/family opinion	Male	21 (13.2)	78 (49.1)	60 (37.7)	0.00
	Female	142 (44.8)	55 (17.3)	120 (37.9)	
Affect family life/ family considerations	Male	29 (18.2)	66 (41.5)	64 (40.3)	0.00
	Female	119 (37.5)	69 (21.8)	129 (40.7)	

*p* < 0.05.

For male students, attracting factors were largely related to the clinical scope of the specialty, including surgical opportunities and caring for a generally healthy patient population. However, sociocultural barriers were prominent deterrents, with patient preference for female physicians, cultural expectations, and spouse or family opinions commonly discouraging interest. Concerns about the impact of OB/GYN practice on family life were also frequently reported among male students.

## Discussion

Achieving a balanced distribution of medical graduates across specialties is essential for maintaining a resilient healthcare system. Obstetrics and gynecology (OB/GYN) remains a specialty facing persistent challenges related to uneven recruitment, particularly across gender. Promoting balanced representation within OB/GYN is increasingly emphasized to ensure workforce sustainability and comprehensive women’s healthcare delivery. In this context, understanding the factors that attract or discourage medical students from pursuing OB/GYN is critical. Accordingly, this study explored medical students’ career intentions toward OB/GYN, along with perceived opportunities, attitudes, and key motivating and deterring factors.

### Career intention of medical students toward OB/GYN

In the present study, approximately 16% of participants identified OB/GYN as their first career choice, a proportion notably higher than that reported in earlier studies, where interest ranged between 3.5 and 8.8% (9, 18, 22, 23). However, around 17% of the participants in our study had considered the specialty as a second choice, which is similar to a study conducted in Jazan, Saudi Arabia (9). Historically, there has been a noticeable decline in the preference for obstetrics and gynecology as the first choice among UK medical graduates from 1974 to 2002. Specifically, the percentage of individuals selecting this field dropped significantly from 4.2% in 1996 to just 2.2% in 1999, which slightly increased in 2002 to be 2.8% (7). According to the results of the research conducted by Ismail and Kevelighan in 2014 and 2020, there has been a consistent or slightly declining trend in pursuing a career in OB/GYN among graduate entry medical students. The percentages associated with this trend were approximately 4 and 3.9% in the respective studies (18, 24). However, In Saudi Arabia, Abu-Rafea et al. (3) reported that only 9.7% of medical students selected OB/GYN as their first choice, highlighting the need for targeted interventions to improve recruitment. This significant increase in the percentage of students choosing the specialty is considered a positive indicator and a first step in opposing the declining trend; however, this increase must be sustained to meet the specialty requirement of medical staff.

A clear gender disparity in career preference was observed, with female students demonstrating significantly greater interest in OB/GYN than male students, consistent with previous national and international findings (8, 25–32). Several factors have been proposed to explain lower male interest, including perceptions of female dominance within the specialty (32), anticipated patient preference for female physicians (33), and limited clinical exposure during OB/GYN rotations due to privacy concerns (34, 35). Male students often report fewer opportunities to perform core examinations, particularly pelvic examinations, which may negatively influence both skill acquisition and specialty perception. Additionally, unfavorable gender bias during OB/GYN clerkships and limited access to leadership roles have been documented (36, 37). These factors likely contribute to the marked decline in male participation in the specialty, as evidenced by the reduction in practicing male OB/GYN physicians from 6.4 to 2.1% between 1990 and 2003 (38). Addressing these barriers is essential, as reliance on female applicants alone may be insufficient to meet future workforce needs.

Interestingly, students’ interest in OB/GYN appeared to decline with advancing academic year, with a striking 81% of 5th-year medical students expressing no interest in the specialty. Students of the 5th year have undertaken their clerkship in OB/GYN, which might contribute to their low interest since students should have interacted with residents and specialists from the specialty. The residents’ attitude toward medical students was not considered as a recruitment factor, as they created an environment as threatening (29). Moreover, students in their 5th year would also have more insight into the lifestyle, which was shown to be one of the most inhibiting factors for students’ interest in OB/GYN (39, 40).

### Opportunities for medical students in OB/GYN

Perceived availability of training and career opportunities plays a substantial role in shaping specialty choice (41). About half of the respondents in our study expressed more opportunities for obstetrics and gynecology training, which correlate to a study conducted at Nottingham University (42). The results of the study indicated that female students were significantly more likely than males to perceive favorable opportunities. These results are similar to the previous findings (43). One potential factor is the increasing representation of females in the medical profession, which may have created a more supportive and inclusive environment for female students pursuing careers in OB/GYN. Additionally, societal stereotypes and gender norms may play a role, as historically, women have been associated with reproductive health and therefore, may feel more encouraged and empowered in these fields. Furthermore, the presence of female role models and mentors in OB/GYN could contribute to the perception of greater opportunity for female students. It is important to note that these factors are not exhaustive and that further research is needed to fully understand the complex dynamics influencing students’ perceptions of opportunity in OB/GYN. Multiple factors can affect males’ opportunities in OB/GYN, including cultural expectations and family opinion, which play a major role (22). Internship status was also associated with perceived opportunities, with interns reporting greater exposure compared to pre-internship students. This may be attributed to increased clinical responsibility, broader procedural involvement, and closer interaction with senior physicians, all of which enhance familiarity with the specialty and confidence in career feasibility.

As expected, our findings showed that married participants had more opportunities for training in OB/GYN than unmarried participants. Dar-Odeh et al. (44) found statistically significant disparities between genders in terms of marital status when choosing a surgical specialty. This suggests that married participants may have had more opportunities for training in surgical specialties compared to unmarried participants. Additionally, another study indicated that the relationship between marital status and specialty training varied for females, while personality preference and work achievement were significant factors for males. This implies that the influence of marital status on specialty training may differ depending on gender (45). For females, factors such as personal interests, preferences, and individual achievements may have played a more significant role in specialty selection compared to marital status. On the other hand, for males, personality preferences and work achievements may have been more influential in determining their choice of specialty. These findings suggest that while marital status can be a factor impacting training opportunities, its significance may vary between genders and be influenced by other factors such as personal interests, achievements, and preferences. This highlights the complex nature of specialty selection and the multiple factors that individuals consider when pursuing specific medical fields. It is worth noting that further research is necessary to fully understand the relationships between marital status, gender, and specialty training opportunities.

### Attitude of splitting of OB/GYN

Interestingly, more than half of participants aged over 25 years expressed support for separating obstetrics and gynecology. Suggesting that greater clinical exposure may influence openness to subspecialization. The results of our study align with another study, where the majority did not agree on splitting (9). There is an increasing trend in favor of splitting both specialties among trainees. The Royal College of Obstetricians and Gynecologists survey indicated a rise in support for splitting from 15% in 1995 to 24% in 1997, further increasing to 34% in 2002 (46). In the Yorkshire Training Programme 2006, this support reached 70% (47). Another report revealed that 55% of specialists were in favor of splitting, although no significant relationship was found (48). Furthermore, many researchers believe that splitting may improve health outcomes and increase the number of sub-specialists. Currently, more than 70% of OB/GYN specialists work as generalists, with an increasing trend towards focusing on either obstetrics or gynecology (49). By splitting the specialties, healthcare professionals can concentrate their expertise on their respective areas, delivering better health services and promoting the development of subspecialties (50). Furthermore, there is evidence suggesting a need to address trainee concerns to retain talent within the specialty. According to Gafson et al. (50), around a quarter of trainees considered leaving the specialty. Splitting the specialty could provide a more tailored career path, potentially increasing job satisfaction and reducing the percentage of trainees considering leaving. A laborist approach has been implemented in a few developed nations in an effort to combat this attrition (51).

### Demographic predictors of career preference for OB/GYN

Multivariable analysis identified gender, university attended, and GPA as significant predictors of interest in OB/GYN. Male students were significantly less likely to express interest, reinforcing the persistence of gender-related barriers despite adjustment for other factors (9, 36, 43, 52–55). Within the Saudi context, this disparity likely reflects cultural norms favoring female physicians in women’s healthcare, limited clinical exposure for male students during OB/GYN rotations, and persistent perceptions regarding patient acceptance and medico-legal risk. The persistence of this association after multivariable adjustment suggests that gender-related perceptions remain a key structural barrier to balanced recruitment. The university attended was also independently associated with interest in OB/GYN. Students from one institution demonstrated higher odds of interest compared with their counterparts, indicating that the institutional environment plays a meaningful role in shaping specialty preference. Differences in clerkship structure, faculty engagement, mentorship availability, and role modeling may explain this variation. Similar institutional effects have been documented internationally, where positive learning climates and supportive faculty interactions during clinical experiences significantly increased student interest in the specialty (56–58). Academic performance, as reflected by GPA, showed a significant association with interest in OB/GYN, with students achieving higher GPAs demonstrating greater odds of expressing interest. This finding aligns with international literature suggesting that academically stronger students are more inclined to pursue specialties perceived as intellectually demanding and clinically rigorous (59–62). In the Saudi setting, where OB/GYN residency positions are competitive and demanding, higher academic achievers may feel more confident in meeting training requirements and managing the specialty’s workload.

### Attracting and discouraging factors

Multiple significant factors were identified as influential in attracting students to OB/GYN. Our results demonstrated that female participants reported that rotation through OB/GYN during medical school, faculty interaction, faculty encouragement/motivation, presence of a role model in OB/GYN specialty, patients’ desire for a female physician, and intellectual content of OB/GYN were found to be significant attractive factors. Regarding the rotation during OB/GYN, faculty interaction, and faculty encouragement/motivation, more than 50% of female participants reported being more attracted towards OB/GYN compared to their male counterparts, as males showed less interest in the specialty through these factors. Multiple studies observed males to be less experienced in terms of practical skills, along with low satisfaction with their own practical experience, and due to gender (36, 46, 52). It is essential to ensure the competency of male medical students’ gynecological practical skills. In fact, good communication skills and empathy are found to be fundamental in these practical skills more than gender (63). Moreover, the disparity in these practical experiences cannot be attributed to patients’ inherent preference for female examiners, as satisfaction with the examination does not exhibit any correlation with the gender of the examiner (64). In contrast, the available evidence suggests that supervisors serve as a formidable impediment to the acquisition of skills in the gynecological examination (65). They possess the ability to actively prohibit male students from participating or passively exclude them, thereby hindering their inclusion in the learning process (65). This study found that both male and female medical students were more likely to consider a career in Obstetrics and Gynecology when they received support and encouragement from faculty members. Specifically, female students were particularly influenced by this interaction. These findings highlight the influence of teaching faculty on both gender during rotations through OB/GYN and during different activities in medical school in developing or sustaining the interest of medical students to pursue OB/GYN as a future career. Similar findings were observed in other studies, reflecting the common occurrence of such factors (3, 66).

Maintaining these attractive factors by concentrating on encouraging teaching faculty to improve their skills in communication with students and to equally interact with medical students of both gender would be a remarkable support in enforcing medical student career pursuit in OB/GYN. Moreover, equal opportunities in different activities should decrease the drawbacks of females compared to males, and expanding exposure will likely be of great assistance.

Our results indicated that the presence of a role model in the OB/GYN specialty is found to be a significantly more attractive factor for females than for males. During clerkship, students interact with specialists, residents, nurses, and midwives. This interaction can play an important role in influencing the decision of students to choose OB/GYN as a future career. Chang et al. (36), observed the professional behavior of residents toward students, providing the opportunity for students to be a part of the team, and offering the chance to gain the practical experience of delivery and labor and, to a minimal extent, with speculum examination was associated with an increase in the interest, and positively influenced the decision to pursue a career in OB/GYN.

Our results also show that males were specifically attracted to the specialty through the surgical opportunities in the field; these results are consistent with other studies (22, 67). This consistency of the result confirms the influence of surgical opportunities in male medical students toward choosing OB/GYN as a future career. Moreover, hands-on opportunities can positively influence the decision to choose the specialty (42). Medical schools should make an effort to maintain the traditional aspect of hands-on experience that is commonly linked to undergraduate OB/GYN curriculum. This is because students frequently consider hands-on experience as a crucial factor in developing their interest and passion in the field (68). Considering these statements, it is vital to encourage male medical students by teaching faculty and residents to perform physical examinations under supervision. Also, engagement of students through a variety of practical activities, such as surgical procedures, early in the course, would improve male medical students’ understanding of the specialty, and the development of clinical judgment and professional interest. Adkins et al. (69), concluded that events that offer early exposure to surgical settings, such as OR essentials, present chances to enhance the confidence of medical students in the operating room. This, in turn, is expected to facilitate the recruitment of aspiring surgeons in the future (69). On the contrary, females were more attracted by the intellectual content of OB/GYN, consistent with previous studies (17, 70). Similarly, the significance found in a study conducted in the central part of Saudi Arabia can confirm the appeal of the specialty by its intellectual content (3).

Conversely, patients’ preference for female physicians, cultural expectations, family influence, and anticipated impact on family life were major discouraging factors for male students, whereas female students more often perceived these factors as attractive. The existing literature certainly indicates the presence of pre-established beliefs about the specialty, and their effect on choosing a career in OB/GYN (17, 26, 71, 72). A misconception exists among male medical students and a significant number of faculty members regarding the preference of female patients for female obstetricians and gynecologists. Although certain demographic groups within the female population may indeed exhibit a strong inclination towards either a male or female physician in this field, the majority of women do not hold a specific preference and instead prioritize establishing a meaningful rapport with their physician (67, 73). Similarities between this study and the aforementioned studies can indicate the possibility of shared cultural beliefs about the specialty. This emphasizes the need to correct these misconceptions and falsely held beliefs about the specialty. Thus, improving the growth rate in the recruitment of males. On the other hand, the level of stress and night duties were the most discouraging factors for female medical students toward the specialty. Lifestyle factors can influence medical students’ career decisions; for this particular reason, it is important to consider these factors cautiously. Newton et al. (41) reported students’ perception of different specialties relating to lifestyle attractiveness; OB/GYN was reported by students to be lifestyle-unfriendly.

This study explored the interest and perceived opportunities of medical students in the specialty, along with addressing different factors that might be linked to this perception. While this study included medical students from all years, it is important to acknowledge several limitations. This study only included medical students from public schools, excluding students from private institutions. Therefore, the results may not fully represent the perspectives of all medical students in Saudi Arabia. Additionally, data were collected from two medical schools located in Eastern Saudi Arabia, which may limit the generalizability of the findings to other academic settings with different curricular structures or training environments. Although stratified sampling by academic year was employed, participant recruitment relied on electronic communication and voluntary survey completion, which may have introduced selection bias, as students who were more engaged or interested in the topic may have been more likely to participate. Moreover, the gender disparity among respondents, with a higher proportion of female participants. Given the established role of sex in shaping perceptions and career preferences in obstetrics and gynecology, this imbalance may have influenced the results and should be interpreted with caution. Another limitation is the reliance on self-reported questionnaire data, which is susceptible to biases such as social desirability bias. Participants may have provided responses they believed to be socially acceptable or in line with expected norms, rather than accurately expressing their genuine views. The use of structured advantages may restrict the exploration of other potential factors, and there is a possibility of response bias affecting the outcomes. Finally, the cross-sectional design of the study precludes any causal inferences; the observed associations should therefore be interpreted as correlational rather than indicative of causal relationships.

## Conclusion

This study highlights the influence of gender-specific factors on medical students’ interest in obstetrics and gynecology. Female students demonstrated a higher inclination toward the specialty, whereas male students encountered barriers related to cultural expectations, family pressures, perceived stress, and limited clinical exposure. Academic performance and institutional factors also shaped career preferences. Strategies aimed at strengthening motivating factors for both genders, addressing misconceptions, enhancing mentorship, and mitigating stress- and culture-related challenges—particularly for male students—may help improve recruitment into the field. Implementing such measures is vital for developing a balanced and sustainable obstetrics and gynecology workforce.

## Data Availability

The original contributions presented in the study are included in the article/supplementary material, further inquiries can be directed to the corresponding author.
